# Validity and reliability of Insomnia Severity Index among older adults in Indonesia

**DOI:** 10.7717/peerj.20473

**Published:** 2026-04-03

**Authors:** Nina Kemala Sari, Stepvia Stepvia, Muhana Fawwazy Ilyas

**Affiliations:** 1 Geriatric Division, Department of Internal Medicine, Dr. Cipto Mangunkusumo National Referral Hospital, Faculty of Medicine, University of Indonesia, Jakarta, Indonesia; 2 Faculty of Medicine, Sebelas Maret University, Surakarta, Central Java, Indonesia

**Keywords:** Insomnia, Insomnia severity index, Validity, Reliability, Indonesia

## Abstract

**Background:**

Insomnia is a prevalent sleep disorder among older adults, associated with fatigue, cognitive decline, and poor physical and mental health. Concise instruments such as the Insomnia Severity Index (ISI) are valuable for screening and evaluation. This study aimed to validate the Indonesian version of the ISI in older adults.

**Methods:**

This study used a cross-sectional survey conducted in multiple health centers and hospitals. The ISI was adapted into Indonesian following the Consensus-based Standards for the Selection of Health Measurement Instruments guidelines. Construct validity was examined using Exploratory Factor Analysis, while reliability was assessed using Cronbach’s alpha and intraclass correlation coefficients (ICC).

**Results:**

A total of 510 older adults (mean age 69.3 years, 81.4% female) participated. Item–total correlations were strong (*r* ≥ 0.75, *p* < 0.01), supporting internal consistency. Factor analysis confirmed a unidimensional structure (Kaiser-Meyer-Olkin = 0.92; Bartlett’s test *p* < 0.01). The ISI showed excellent reliability (Cronbach’s alpha = 0.96; ICC = 0.96).

**Conclusion:**

This study demonstrates that the ISI is a valid and reliable instrument for evaluating perceived insomnia severity among older adults in Indonesia. Its ease of use and strong psychometric properties make it an invaluable tool for healthcare practitioners in routine screening and clinical practice.

## Introduction

The global population of older adults is experiencing a remarkable surge, increasing from approximately 205 million individuals today to an anticipated two billion by the year 2050 (Patel, Steinberg & Patel, 2018). In 2019, individuals aged 60 years and above constituted 9.7% of Indonesia’s total population, equivalent to approximately 26 million individuals (Budiman et al., 2024). Insomnia is one of the most common sleep disturbances among older adults. It is often characterized by the subjective experience of difficulties in beginning or sustaining sleep, or experiencing non-restorative sleep, leading to considerable daytime symptoms such as impaired concentration and mood disturbances. The global prevalence of insomnia complaints among older adults varies from 30% to 48% (Patel, Steinberg & Patel, 2018). Subsequently, the prevalence of insomnia among older adults in Indonesia has been reported to range from 22% to 36.7% (Bangun, Gondodiputro & Andayani, 2020; Budiman et al., 2024). A previous study also demonstrated that the prevalence of insomnia is markedly elevated among older adults with lower levels of education, poor health, overweight or obesity classifications, diminished self-satisfaction, feelings of loneliness, dependence, and depression (Handajani et al., 2024).

Insomnia in older adults can significantly impact sleep quality, leading to daytime fatigue, cognitive impairment, and a decline in both physical and psychological health. It can also decrease the overall quality of life. Studies have identified correlations between insomnia and numerous medical and psychological conditions, including heightened susceptibility to psychiatric disorders, suicide, and chronic health difficulties such as obesity, diabetes, cardiovascular disease, and chronic pain. Therefore, if left untreated, insomnia can result in considerable morbidity (Nguyen, George & Brewster, 2019; Bangun, Gondodiputro & Andayani, 2020).

The evaluation of insomnia is a multifaceted approach that ideally encompasses a clinical assessment, self-report questionnaires, and daily sleep diaries. Although clinical examination is the gold standard for accurately diagnosing insomnia, it can be time-consuming in regular clinical practice. This may discourage some healthcare practitioners from systematically addressing sleep issues with all their patients. Concise and reliable questionnaires can significantly aid in the preliminary assessment and formal evaluation of insomnia. The Insomnia Severity Index (ISI) is a succinct instrument intended to evaluate the severity of insomnia symptoms, both nocturnally and diurnally. The ISI is available in multiple languages and is increasingly utilized as a measure of therapy response in clinical research. Moreover, the ISI offers critical insights for evaluating the importance and therapeutic implications of changes in insomnia symptoms (Morin et al., 2011).

The Insomnia Severity Index has been commonly utilized in Indonesia to evaluate insomnia; nevertheless, there are no specific findings concerning its application among older adults. This study aimed to evaluate the validity and reliability of the Indonesian version of the ISI among older adults in Indonesia, considering its accessibility, convenience, and psychometric properties.

## Materials & Methods

### Study design

This was a cross-sectional study designed to evaluate the psychometric properties of the Indonesian version of the ISI in older adults. This study complied with the COSMIN (Consensus-based Standards for the selection of health measurement instruments) guidelines for instrument validation (Mokkink et al., 2016). Subsequently, the reported features in this study are guided by the STROBE (Strengthening the Reporting of Observational Studies in Epidemiology) checklist (Von Elm et al., 2007). Ethical approval was obtained from the Faculty of Medicine, University of Indonesia—Dr. Cipto Mangunkusumo National Referral Hospital on January 6, 2025 (Ethical Application Ref: 24-10-1617). All participants provided written informed consent before participation.

Data collection was performed from January 10, 2025 to February 28, 2025 at the Ciketing Udik Primary Health Center (Puskesmas Ciketing Udik), Bekasi, Indonesia; Paris Integrated Community Health Service Post (Posyandu Paris), Bogor, Indonesia; Senior Expo, Jakarta, Indonesia; and Dr. Cipto Mangunkusumo National Referral Hospital, Jakarta, Indonesia. The sample size was determined according to widely accepted recommendations for factor analysis, which suggest a minimum of 5–10 participants per questionnaire item. As the ISI comprises seven items, a sample of at least 70 would have been sufficient. Subsequently, 600 older adults aged 60 years and older were recruited using purposive sampling based on eligibility criteria. This approach was chosen to ensure the inclusion of older adults from both community and hospital settings. Participants were required to be fluent in Bahasa Indonesia and capable of providing informed consent. Individuals with cognitive impairments, as identified through a brief cognitive screening conducted by trained researchers and clinical judgment, were excluded. Significant medical conditions known to interfere with sleep including untreated sleep apnoea, advanced neurological disorders, or severe psychiatric illness were also considered exclusion criteria. Participants currently taking medications that substantially influence the sleep–wake cycle were also excluded.

### Translation and cultural adaptation of the ISI

The ISI is a seven-item questionnaire intended to assess nocturnal symptoms and the effects of insomnia experienced in the preceding month. Scores on this scale span from 0 to 28, with values exceeding 14 signifying moderate to severe insomnia. The questionnaire covers difficulties with sleep onset, sleep maintenance, and early awakening, as well as the impact of insomnia on daily functioning and overall satisfaction with sleep (Brewster, Riegel & Gehrman, 2017; Cerri et al., 2023). In this study, the original ISI was translated and culturally adapted for the Indonesian population following COSMIN-recommended procedures. Two bilingual translators, fluent in English and Bahasa Indonesia and knowledgeable in sleep medicine, independently translated the ISI into Bahasa Indonesia. These translations were reviewed and synthesized into a single version by a panel of experts in sleep-related health. A back-translation was then performed by two independent translators who were blinded to the original ISI. The back-translated version was compared to the original to ensure semantic and conceptual equivalence, with discrepancies resolved by consensus.

The pre-final version was pilot-tested on 30 older adults at Dr. Cipto Mangunkusumo National Referral Hospital in Jakarta to assess clarity, cultural appropriateness, and comprehension. Feedback from this pilot phase indicated minor issues with wording clarity and cultural appropriateness, particularly in items related to daily functioning and satisfaction with sleep. These were adjusted to improve comprehension, and the refined version was then finalized as the Indonesian ISI. Further, the final full Indonesian version of the ISI used in this study is provided in Table 1 under the Creative Commons Attribution (CC BY) license. The original English version is publicly available and widely used in clinical and research settings. All necessary permissions have been obtained, and the translated version is published with proper attribution to the original developers.

**Table 1:** ISI item responses among participants.

ISI Questions	Median (IQR)
Q1 - *Kesulitan untuk tertidur* [Difficulty falling asleep]	0 (0–2)
Q2 - *Kesulitan untuk tetap tertidur* [Difficulty staying asleep]	1 (0–2)
Q3 - *Masalah bangun terlalu awal* [Waking up too early]	0 (0–2)
Q4 - *Seberapa****PUASKAH****/ tidak puaskah Anda dengan pola tidur Anda saat ini?* [Satisfaction with current sleep pattern]	1 (0–2)
Q5 - *Sejauh mana Anda menganggap masalah tidur Anda****MENGGANGGU****fungsi harian Anda (misalnya, kelelahan di siang hari, kemampuan bekerja/melakukan tugas harian, konsentrasi, memori, suasana hati)?* [Impact of sleep problem on daily functioning]	0 (0–2)
Q6 - *Seberapa****TERLIHAT****bagi orang lain menurut Anda masalah tidur Anda dalam hal mengganggu kualitas hidup Anda?* [Perceived impact of sleep problem on quality of life]	0 (0–2)
Q7 - *Seberapa****KHAWATIR****/ tertekan Anda tentang masalah tidur Anda saat ini?* [Worry/distress about current sleep problem]	0 (0–2)
Total (sum of all items)	3 (1–12)

### Validity and reliability testing

Data were gathered from participants to assess the psychometric features of the Indonesian ISI. The questionnaire was administered in a paper-based format under the supervision of trained researchers at the study sites to ensure accurate responses and understanding. To enhance the reliability of data collection, standardized instructions were provided to participants, and researchers remained available to clarify any ambiguities in the questions. Thereafter, test-retest reliability was evaluated using a subset of 50 randomly chosen participants who completed the ISI a second time following a two-week gap. This approach ensured consistency in responses over time and evaluated the stability of the instrument.

### Statistical analysis

Statistical analyses were conducted using IBM SPSS Statistics version 27.0 (IBM Corp., Armonk, NY, USA). Descriptive statistical analyses were performed for continuous variables as means and standard deviations (SD) or median and interquartile range (IQR), while categorical variables were reported as frequencies and percentages. Construct validity was evaluated through Exploratory Factor Analysis (EFA), using the Kaiser-Meyer-Olkin (KMO) measure (> 0.5) to assess sampling adequacy and Bartlett’s test of sphericity (*p* < 0.05) to confirm factorability. Factors with eigenvalues exceeding 1 were retained, and the scree plot was reviewed to confirm the factor structure. Reliability was subsequently evaluated by internal consistency with Cronbach’s alpha, with values of ≥ 0.7 considered acceptable. Additionally, intraclass correlation coefficients (ICCs) were calculated, with values ≥0.75 indicating excellent reliability. Last, gender differences in ISI categories were examined with Chi-square tests and, where appropriate, the Kolmogorov–Smirnov test. A *p*-value below 0.05 was considered statistically significant for all analyses.

## Results

### Selection of the research participant

**Figure 1:**
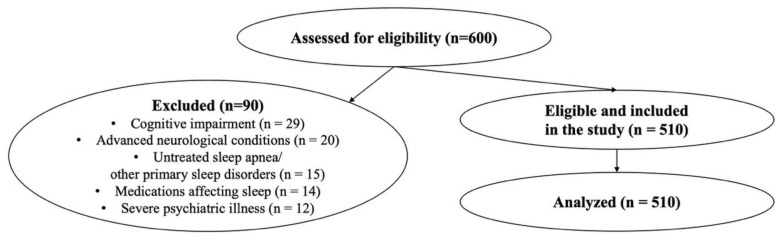
Flow diagram of study participant selection and analysis.

A total of 600 older adults were screened for eligibility. Of these, 90 did not meet the inclusion criteria and were therefore excluded. The primary reasons for exclusion were as follows: 29 individuals demonstrated evidence of cognitive impairment on brief screening or clinical judgment. Twenty participants reported advanced neurological conditions such as Parkinson’s disease or dementia. Fifteen were identified as having untreated sleep apnoea or other primary sleep disorders requiring specialist evaluation. Fourteen were receiving medications with significant effects on sleep architecture, including high-dose sedatives and corticosteroids. Additionally, twelve individuals were found to have severe psychiatric illness, predominantly major depressive disorder with active symptoms. After these exclusions, the final analytic subjects comprised 510 participants who met all eligibility requirements (Fig. 1).

### Socio-demographic characteristics of the research participants

A total of 510 participants were included in this study. Table 2 indicates that the majority of participants were female (415, 81.4%), with a mean age of 69.27 years (SD = 6.62). Regarding education, most respondents held an associate degree (152, 29.8%), while 125 (24.5%) had completed senior high school. In terms of living arrangements, the majority of participants (371, 72.7%) resided with their spouses. Subsequently, Table 1 presents the distribution of responses to each item of the ISI. The median total ISI score was 3 (IQR 1–12). The majority of participants (67.8%, *n* = 346) exhibited clinically insignificant insomnia (ISI scores 0–7), while 11.6% (*n* = 59) had subthreshold insomnia (ISI scores 8–14), 9.2% (*n* = 47) had clinical insomnia of moderate severity (ISI scores 15–21), and 11.4% (*n* = 58) experienced severe clinical insomnia (ISI scores 22–28) (Table 3).

### Validity of the questionnaire

**Figure 2:**
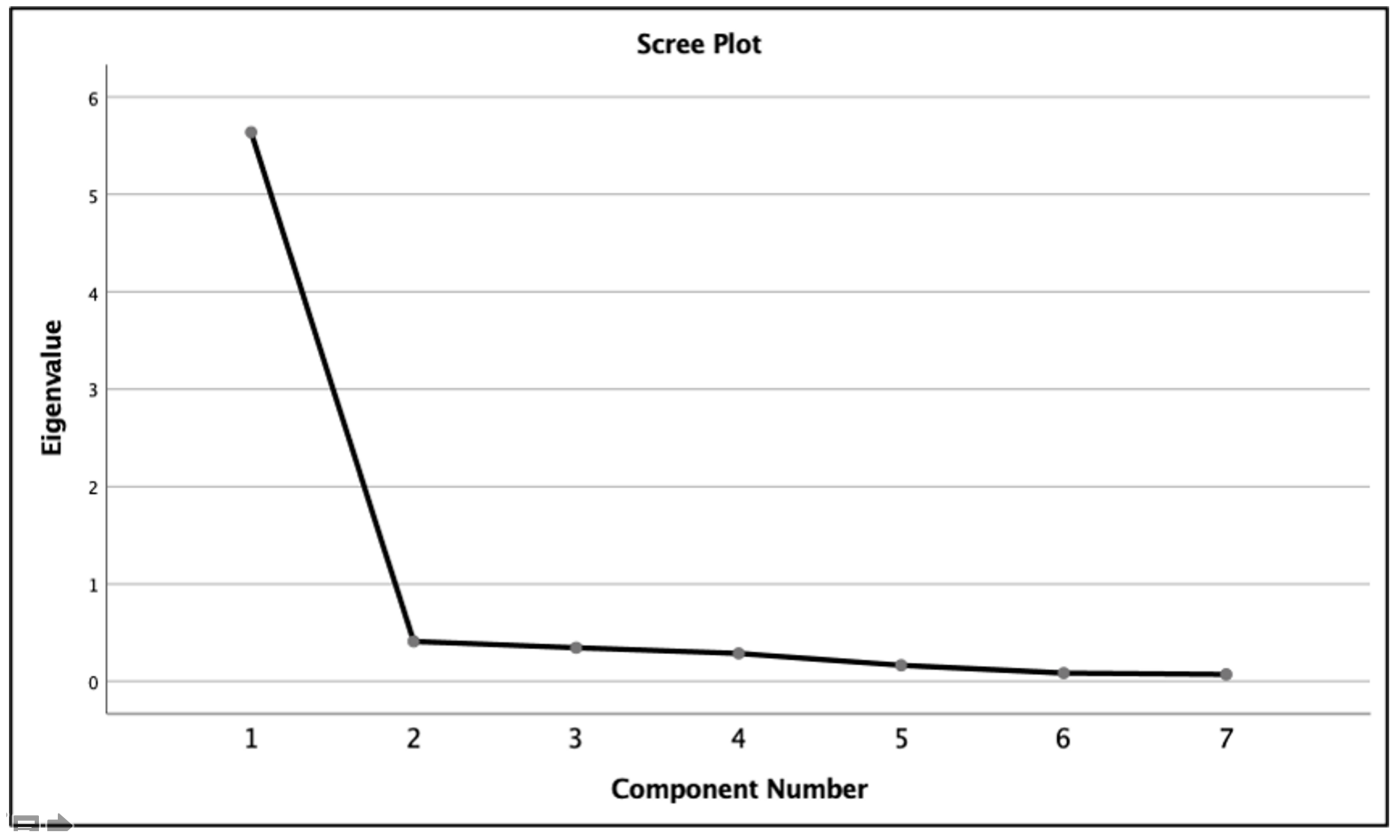
Scree plot of factor loadings for the ISI in the study population (retained factor with eigenvalue = 5.64, explaining 80.55% variance).

The ISI demonstrated strong validity metrics. Content and face validity were established through consensus among panelists. Spearman’s Rank-Order correlation analysis revealed substantial relationships between each item and the total ISI score, with all correlation coefficients ≥ 0.75 (*p* < 0.01), as shown in Table 4. Subsequently, construct validity was confirmed through EFA. Bartlett’s test of sphericity demonstrated that the correlation matrix was suitable for factor analysis (*x*^2^_28_ = 4,250.99, *p* < 0.01), and the KMO measure of sampling adequacy was excellent at 0.92. A single-factor model was retained based on the initial eigenvalue of 5.64 and the unidimensional latent structure observed in the scree plot (Fig. 2). The retained factor explained 80.55% of the total variance, further supporting the validity of the ISI for this population.

**Table 2:** Socio-demographic characteristics of study participants (*N* = 510).

Sample	Characteristics	n (%) (*N* = 510)
Sex	Male	95 (18.6%)
	Female	415 (81.4%)
Age (years)		Mean (SD): 69.27 (6.67)
Education	No formal education	9 (1.8%)
	Elementary school graduate	66 (12.9%)
	Junior high school graduate	21 (4.1%)
	Senior high school graduate	125 (24.5%)
	Associate graduate	152 (29.8%)
	Bachelor’s degree	102 (20.0%)
	Master’s degree	30 (5.9%)
	Doctorate degree	5 (1.0%)
Residing with	Spouse	371 (72.7%)
	Sibling(s)	8 (1.6%)
	Relative(s)	2 (0.4%)
	Child(ren)	88 (17.3%)
	Grandchild(ren)	2 (0.4%)
	Caregiver	3 (0.6%)
	Living alone	23 (4.5%)
	Others	13 (2.5%)

**Table 3:** Distribution of ISI categories among participants.

Category	N (%)
No clinically significant insomnia (0–7)	346 (67.8%)
Subthreshold insomnia (8–14)	59 (11.6%)
Clinical insomnia (moderate severity) (15–21)	47 (9.2%)
Clinical insomnia (severe severity) (22–28)	58 (11.4%)

**Table 4:** Spearman’s rank-order correlation between each item and total score.

Questions	*r*	*p*-value
Q1	0.75	<0.01
Q2	0.84	<0.01
Q3	0.79	<0.01
Q4	0.88	<0.01
Q5	0.81	<0.01
Q6	0.83	<0.01
Q7	0.84	<0.01

### Reliability of the questionnaire

The ISI demonstrated excellent reliability. Internal consistency was assessed using Cronbach’s alpha, yielding a value of 0.96, signifying a high level of consistency among items. The ICCs were also excellent at 0.96 (95% CI [0.95–0.96], *p* < 0.01), demonstrating the stability and reliability of the questionnaire over time (Table 5). Subsequently, test-retest reliability, evaluated in a sample of 50 subjects who completed the ISI twice with a two-week gap, further confirmed the tool’s stability. These findings indicate that the ISI provides consistent results when administered repeatedly under similar conditions.

**Table 5:** Internal consistency and intraclass correlation coefficient (ICC).

Question items	Cronbach’s alpha	r (ICC)	95% CI	*p*-value
Overall (Q1-Q7)	0.96	0.96	0.95–0.96	<0.01^*^

**Notes.**

* Statistically significant at *p* = 0.05.

Gender-based analysis of ISI categories indicated no statistically significant variations in insomnia severity between males and females (*p* = 0.54). While females were numerically more frequent in each ISI category (reflecting the larger female sample), particularly in the severe clinical insomnia group (11.6% *vs.* 10.5% for males), these disparities did not attain statistical significance (Table 6). The data indicate that the distribution of insomnia severity, as classified by the ISI, was similar across genders in this population.

**Table 6:** Gender differences in ISI categories.

Questions	Males (N, %)	Females (N, %)	*p*-value
No clinically significant insomnia	60 (63.2%)	286 (68.9%)	0.54
Subthreshold insomnia	14 (14.7%)	45 (10.8%)	
Clinical insomnia (moderate severity)	11 (11.6%)	36 (8.7%)	
Clinical insomnia (severe severity)	10 (10.5%)	48 (11.6%)	

**Notes.**

Analyzed using Chi-square test.

## Discussion

The ISI is a concise assessment tool designed to aid healthcare professionals in evaluating patients who report insomnia-related issues. It is also utilized to measure the effectiveness of treatment interventions in clinical research settings. Comprehensive research has established the validity and reliability of the ISI, confirming its efficacy in various populations. Our research sought to rigorously examine the validity and reliability of the ISI in its Indonesian version, especially for older adults. The findings from our study provide strong support for the questionnaire, demonstrating that it is both a valid and reliable tool for screening insomnia among older adults in Indonesian-speaking populations. By including 510 participants, our study far exceeded the minimum requirement of sample size, ensuring adequate statistical power and strengthening the reliability of the results.

This study demonstrated that Spearman’s Rank-Order correlation analysis produced high correlation coefficients, indicating strong associations between the ISI items and the total score. The construct validity analysis indicates favorable outcomes which imply the factorability of the correlation matrix. The Kaiser-Meyer-Olkin test also indicates sufficient sampling in relation to the number of questionnaire items. The Cronbach’s alpha coefficients indicated a high internal consistency and the ICC analysis demonstrated an acceptable outcome, reflecting excellent reliability for the Indonesian version of ISI. Although this very high Cronbach’s alpha confirms excellent internal consistency, it may also suggest potential redundancy among items, a point that warrants further exploration in future studies. The validity and reliability measurement in this study aligns with previous studies that reported high internal consistency coefficients, particularly with Cronbach’s alpha values ranging from 0.65 to 0.92. Most of these studies noted values exceeding 0.70, indicating the ISI’s strong reliability (Cho, Song & Morin, 2014; Veqar & Hussain, 2017; Chahoud et al., 2017a; Manzar, Jahrami & Bahammam, 2021; Cerri et al., 2023).

The ISI has proven to be a valuable tool in primary care settings, effectively identifying individuals who experience clinically significant insomnia. Insomnia severity in older adults is affected by various factors, including demographic variables such as living in urban *versus* rural areas, as well as psychological aspects like depression and anxiety. Older adults often also face more difficulties with maintaining sleep compared to younger adults. Healthy older adults commonly experience an advanced sleep phase, meaning they tend to fall asleep and wake up early. However, this pattern may not apply to older adults who experience insomnia symptoms, as they may have a delayed circadian phase (Brewster, Riegel & Gehrman, 2017; Brouwer et al., 2022). Additionally, physical health conditions play a critical role in exacerbating sleep disturbances among older adults (Gagnon et al., 2013; Liao et al., 2024).

In the current study, most participants were female (81.4%). This gender imbalance may have influenced the findings, as females often report higher rates of insomnia than males (Zeng et al., 2020), and thus the results may not fully reflect the experience of male older adults. The relatively high level of education among many participants, with nearly one-third holding an associate degree, could also have affected questionnaire comprehension and response accuracy, potentially leading to better psychometric performance of the ISI than in populations with lower literacy. Additionally, the fact that most respondents lived with their spouses (72.7%) suggests a supportive environment, which might mitigate some of the negative impacts of insomnia compared to those living alone. These demographic features should be considered when interpreting the results, and future research should aim to recruit more balanced samples in terms of gender, education, and residential background to increase generalizability.

Overall, the ISI stands out as an effective screening instrument for healthcare providers working with older adults, allowing them to identify individuals who may benefit from further evaluation or targeted interventions. It provides a comprehensive assessment of insomnia by capturing difficulties with sleep onset, maintenance, and early awakening, as well as the impact of these symptoms on daily functioning and quality of life. This makes it possible for clinicians to distinguish between different levels of insomnia severity and tailor management strategies accordingly. Moreover, integrating the ISI into community health programs and routine geriatric assessments in Indonesia could improve early detection of insomnia and help reduce associated morbidity.

This study has important strengths, including a relatively large sample drawn from multiple healthcare facilities, but several limitations should also be acknowledged. Because of its cross-sectional design, the study cannot establish causality or assess changes in insomnia severity over time; this means that while the ISI showed good psychometric properties at a single point, its stability across different stages of ageing or over longer periods remains uncertain. Second, the study relied on self-reported data, which is susceptible to recall bias and subjective interpretation. While the ISI is a validated instrument, the inclusion of objective measures such as actigraphy or polysomnography could strengthen future studies. Subsequently, the sample is not balanced in terms of gender and setting (81% female, predominantly urban), which limits the generalisability of the results and should be highlighted in the limitations of the study. Finally, as purposive sampling was used, the sample may not fully represent the broader population of older adults in Indonesia. This non-probability method could therefore reduce external validity, particularly for underserved or remote populations. Future longitudinal studies, incorporating both subjective and objective sleep measures, are needed to better establish the ISI’s responsiveness and predictive validity in diverse Indonesian older adult populations.

## Conclusions

This research illustrates that the Indonesian version of the ISI is a valid, reliable, and effective instrument for evaluating insomnia severity in older adults. Its concise format and user-friendly design allow healthcare providers to administer it efficiently in clinical and community settings. Despite potential variations in interpretation due to cultural, educational, or gender differences, the ISI has demonstrated consistent psychometric strength across diverse populations. Future research employing longitudinal and objective measures is recommended to further support its use and enhance generalizability.

##  Supplemental Information

10.7717/peerj.20473/supp-1 Supplemental Information 1 Statistical Input and Output

## Additional Information and Declarations

### Data Availability

The following information was supplied regarding data availability: The raw data is available in the Supplemental Files.
